# Research on risk contagion mechanism of big fintech based on the SIRS model

**DOI:** 10.1371/journal.pone.0291230

**Published:** 2023-09-08

**Authors:** Yutong Li, Zhongming Tan, Chenyu Huang

**Affiliations:** School of Finance and Economics, Jiangsu University, Zhenjiang, Jiangsu, China; Zhejiang Gongshang University, CHINA

## Abstract

In recent years, while the relationship between the new financial institutions, represented by financial technology companies, and the traditional financial institutions(banks, securities, insurance, etc.) has been steadily enhanced, a New Relational Network has silently emerged. Along with the rapid expansion of big fintech companies, the possibility of financial risk breeding and spreading in the New Relational Network is also rising. This article analyzes and simulates the risk contagion mechanism of big fintech risks based on the SIRS epidemic model. The study’s findings imply that: when the number of big fintech companies infected with risk exceeds the risk threshold, the big fintech risk will spread in the New Relational Network. At this time, the number of big fintech companies infected with risk can be reduced below the threshold by enhancing the risk warning, risk management, risk buffering and blocking capabilities, and timely improving risk prevention and control measures in the post-infection phase. It means that the big fintech risk is controlled. For big fintech risks, proactive interventions are more effective than post-incident response measures. This paper makes the following recommendations for preventing big fintech risks: creating a risk monitoring and early warning system to raise the Big Fintech companies’ direct immunization rates; strengthening the big fintech companies’ risk management and risk mitigation capabilities; enhancing the internal and external supervision to achieve sustainable development of big fintech companies.

## 1 Introduction

With the widespread use of emerging technologies such as artificial intelligence, blockchains, and big data in the financial field, fintech has emerged. The term "FinTech" originated in the United States in the 1990s and attracted widespread attention in academia after being officially proposed in 2011. Financial technology (FinTech) is defined by the Financial Stability Board (FSB) as "technology-driven financial innovation", which applies a series of advanced scientific and technological achievements, such as big data, blockchain, artificial intelligence, and so on to the financial field, emphasizes the auxiliary, supporting and optimizing role of new technologies in financial business [1], and gradually achieves integrated development. While promoting financial innovation, new financial institutions represented by fintech companies have quietly formed a New Relational Network with traditional financial institutions (banks, securities, insurance, etc.), and various financial risks are transmitted and overflowed through the associated path.

In recent years, some big technology companies (BigTech) with mature technology have become involved in the financial sector with their technology, capital, flow, brand, and other advantages. These companies have gradually developed the business model and service ecology of "BigTech in Finance," bringing disruptive changes to traditional finance. Because of its vital role in promoting the deep integration of new technologies and finance, Big Tech has received a lot of attention. Some news media initially adopted the concept of Big Tech for example, Google, Amazon, Facebook, and Apple as the "Big Four Tech" and later deleting the middle quantifier and calling it "Big Tech" instead [2]. In August 2017, the World Economic Forum (WEF) and Deloitte jointly published a research report titled "Beyond Fintech: A pragmatic assessment of disruptive potential in financial services", which directly followed the media approach and specifically referred to BigTech as Google, Apple, Facebook, and Amazon (GAFA) in the United States and Baidu, Alibaba, Tencent, and JD.com (BATJ) in China [3]. Big Tech companies have also become more and more active in the financial services sector in other regions. Examples include Kakao Bank in South Korea, Line in Japan, and Go-Jek in Southeast Asia [4]. In October 2017, the Basel Committee on Banking Supervision (BCBS) published a working paper, " Sound Practices: Implications of Fintech Developments for Banks and Bank Supervisors ", which for the first time defined Big Tech from the official perspective of international organizations, and believed that Big Tech is a global large-scale Internet information technology platform with an absolute advantage in digital and technology [5]. Therefore, the big fintech companies mentioned in this article are "Big Tech companies that enter the financial field and participate in financial business with their technological, user, and platform advantages ".

Financial networks, as opposed to other network types, typically exhibit the traits of scale-free networks, that is, a few numbers of large nodes (Represented as systemically important financial institutions in the financial field) have a great number of connections while most tiny nodes only have a small number of connections. Due to their high market concentration and sizable business scale, these big fintech companies have gradually become large nodes in the New Relational Network, which also highlights the hidden risks of big fintech companies—in the case of a large number of connections, once risks arise, they are easy to infect other financial entities through related paths such as business, investment and financing, and public expectations [6], thus triggering systemic financial risks. It can be seen that, as an emerging financial format, big fintech companies not only serve the long tail of the population and enhance user experience, but make financial hazards more contagious [7]. Therefore, it is of great significance to deeply explore the contagion process and transmission mechanism of big fintech risks and study the prevention strategies of big fintech risk contagion.

The majority of research on the types of fintech risks is qualitative at the moment because of challenges with data collection. As an extension of fintech risks, big fintech risks not only include operational risks of traditional financial institutions, but also risks of Internet financial institutions and technology companies [8–12]. As a result of fintech’s rapid development, the financial system is now more correlated, market swings are amplified, and risk contagion is increased [12, 13].

Big fintech risks, in contrast to traditional fintech risks, also have their distinctive new risks [14]: first, monopoly risks. Big fintech companies have a significant degree of market concentration as a result of their enormous user and business scales; meanwhile, the platform effect also implies potential monopoly risk, and market monopoly is looming [4, 15, 16]. Procyclical risk is the second. The AI algorithms used by different big fintech companies are quite similar, which causes the risk management, investment, and financing policies to converge and magnifies procyclical asset fluctuations [12, 17]. Liquidity risk is the third. The application of new technologies has given the customers of big fintech companies the freedom to make more profitable choices between different deposit accounts and mutual funds, increasing liquidity risk [18]. Fourth, high leverage risk. When cooperating with traditional financial institutions, big fintech companies use technology platforms to quickly acquire customer flow with low customer acquisition costs and sell pure credit financial products without collateral endorsement to customers, which increases the personal debt ratio and does not inject their own funds, exacerbating the problem of high leverage [18, 19]. Fifth, it exacerbates financial systemic risks. The position of big fintech companies in the financial network, which is considered systemically important, increases systemic financial risk. Big fintech companies’ monopolistic market positions increase their competitive advantage in the financial business and increasingly draw attention to their attributes as quasi-public goods. Other financial institutions will be impacted when such big fintech companies with financial infrastructure characteristics breed risks. Due to their "too big to fail," "too connected to fail," and "too broad to fail" status, the government will also step in and provide assistance even in times of crisis, which makes big fintech companies even more fearless and gives rise to moral hazard [19, 20]. Financial stability is threatened by each of these risks.

In recent years, due to the high similarity between financial risk contagion and the spread of medical infectious diseases [21], some scholars have studied financial risk contagion from the perspective of epidemic transmission. In order to study the spread of the Black Death in London, Kermack and McKendrick proposed the famous SIR model in 1926. The SIR model is appropriate for infectious diseases for which recovery leads to lifetime immunity. The SIR model has further developed into models like SI and SIS because infectious diseases can manifest in a variety of ways in reality. Garas et al. (2010) introduced the epidemic contagion mechanism when studying the contagion of the global economic crisis, and used the SIR Model to simulate the process of crisis contagion [22]; Toivanen (2013) and the Research Group of PBC Guangzhou Branch (2021) introduced the infectious disease model into the study of inter-bank financial risk contagion [23, 24]; Haldane (2013) found that the risk contagion caused by the bankruptcy of Lehman Brothers was similar to the spread of SARS [25]; In order to study the cross-contagion mechanism of financial market risks, the Research Group of PBC Nanning Branch (2017) used the SIRS epidemic model [26]; Wang et al. (2017) built an epidemic model of default public opinion and examined P2P creditor’s rights transfer mode and default public opinion contagion mechanism [27]; Xu et al. (2018) used SIRS model to construct the risk contagion model of mutual guarantee network of small and medium-sized enterprise clusters [28]; Mi et al. (2019) conducted a simulation analysis of Internet financial risk contagion based on the SEIS model [29]. Therefore, the application of the epidemic model to the financial field provides a new perspective for studying the risk contagion mechanism of big fintech.

While improving financial efficiency and promoting financial development, big fintech companies controlled by platform capital also have huge risks and hidden dangers [16]. BATJ and some technology companies have been interviewed by Chinese regulatory authorities, which has attracted widespread attention from the academic community. However, the mechanism of risk contagion in big fintech has not yet been fully understood, and research on related theories has just begun. The contribution of this article is reflected in:(1) By researching their distinct risk kinds and overall risk contagion, big fintech companies—a new form of the financial institution that integrates finance and technology—can enhance previous theoretical accomplishments. (2) With a focus on the big fintech risk spreading mechanism, this paper simulates and studies the big fintech risk transmission characteristics using the SIRS epidemic model. (3) It is possible to improve the understanding among relevant financial organizations and regulatory authorities about big fintech risks and to preserve the overall goal of avoiding systemic financial risks by studying the contagion laws of big fintech risks and simulating the risk contagion processes.

The structure of this paper is as follows: In Section 2, we build a big fintech risk contagion model based on the SIRS model; The Section 3, we simulate and analyze the model; The Section 4, we put forward some targeted suggestions based on the above analysis results.

## 2 Model construction and analysis

### 2.1 Contagion mechanism and model selection

**Fig 1:**
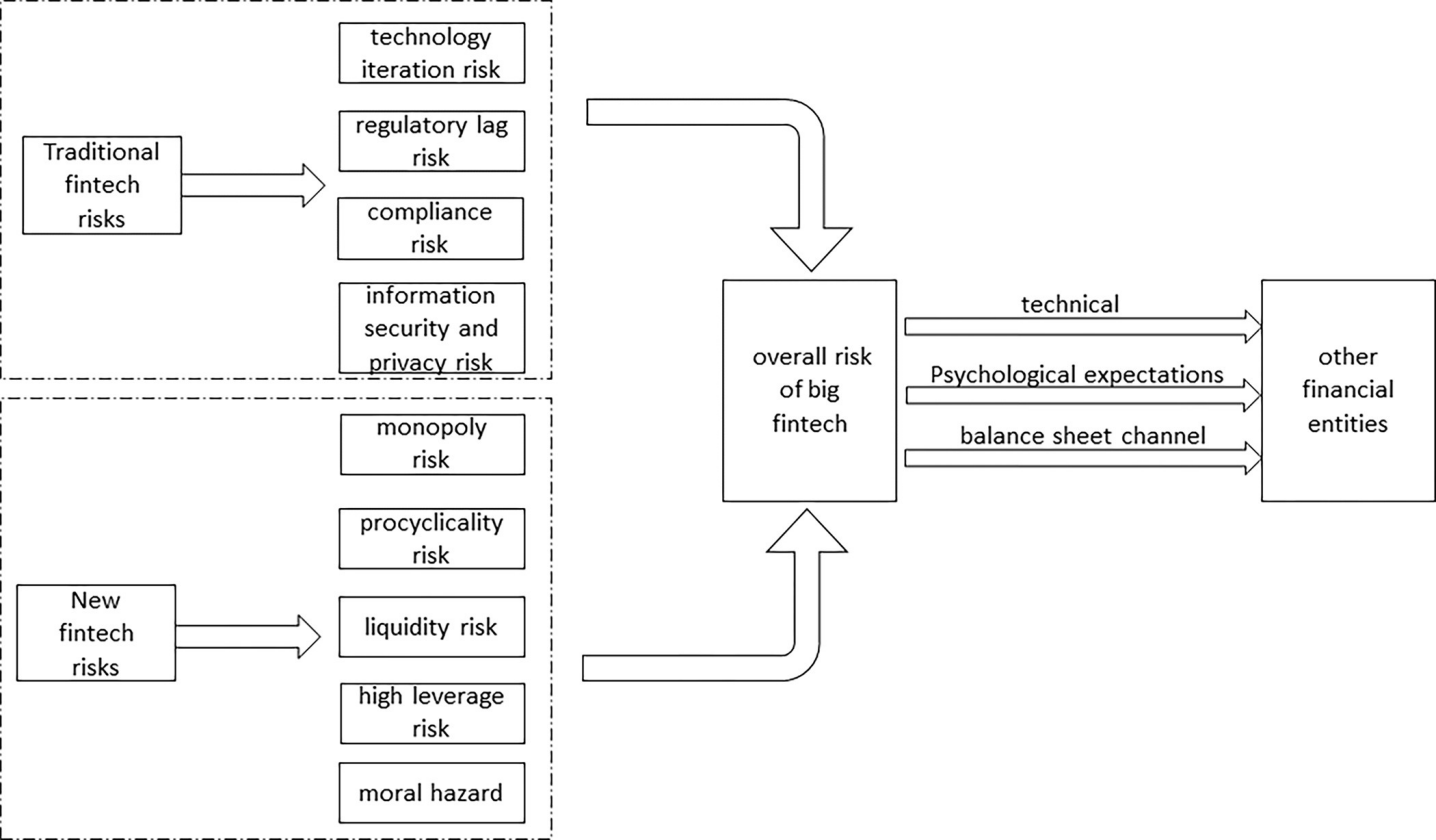
Risk contagion channel of big fintech.

Although technology by itself does not bring financial risks, its application to financial operations will do so [19, 30]. According to the previous analysis, big fintech risks not only include traditional fintech risks such as technology iteration risk, regulatory lag risk, compliance risk, information security, and privacy risk, but also include new risks such as monopoly risk, procyclicality risk, liquidity risk, high leverage risk, and moral hazard. These risks affect and correlate with each other, forming the overall risk of big fintech. The occurrence of a single risk may generate an overall risk in the context of the increasingly interconnected situation of different financial institutions [31], which can be transmitted to other financial entities through the channels depicted in Fig 1: The first is the technical channel. Big fintech companies have a relatively high level of system connection [12], which raises the correlation between financial institutions and the sensitivity of financial activities. The financial operations of one big fintech company can have an impact on other big fintech companies. Secondly, through customer anxiety and psychological expectations channel [32]. Big fintech companies primarily serve the long-tail market, where individual customers are unable to make professional investment decisions. Herd psychology, which results in the herd effect, causes individual illogical actions to lead to group irrational decisions. Investors will lose confidence in other big fintech companies once a big fintech company faces risks. The balance sheet channel comes in third. Big fintech companies establish bilateral debt relationships with other financial institutions through the money market or capital market. If a financial institution generates risks that lead to a deterioration in its asset conditions, those risks can be transmitted to other financial institutions via the balance sheet channel.

At present, the research methods for studying the mechanism of risk contagion mainly include canonical correlation analysis (CCA), Copula function method, Granger causality analysis, infectious disease models [21, 22, 33–36], etc. After initial clarification of the contagion mechanism of big fintech risks, it is evident that the contagion process of big fintech risks is similar to the spread of epidemics such as SARS and COVID-19. Therefore, it is more suitable to examine the contagion mechanism of big fintech risks using an appropriate infectious disease model. The reasons are as follows:

1. In the New Relational Network, many individuals are exposed to risk and are susceptible to risk. Different individuals have differences in risk management level, asset status, liquidity level, and other aspects, resulting in different individual situations. Individuals with strong anti-risk ability are less susceptible to risk contagion, while individuals with poor anti-risk ability are more prone to risk. This is the basis for using the contagion model to study the contagion mechanism of big fintech risks.
2. When risks arise, due to the high market concentration, large business scale, and intensive connection between big fintech companies and other financial institutions, these risks are easily transmitted to other financial institutions through risk carriers like business associations and public expectations. Even if a big fintech company experiences a risk event without direct business associations, it can still transmit the risk to other big fintech companies through public expectations. This leads to a risk contagion "multiplication effect" in the New Relational Network, similar to the concept of "super-spreaders" in the transmission of infectious diseases.
3. When a risk occurs, it will spread rapidly in the New Relational Network due to the presence of association channels, consistent with the features of the transmission of infectious diseases in a population. The recovery and recurrence after risk infection are also present in big fintech companies. However, the risk removal does not imply permanent immunity, as there may be other channels of risk contagion that could lead to the risk of reinfection, similar to the transmission characteristics of infectious diseases within a population.

Therefore, based on the SIR Model, considering that the risk removal only provides temporary immunity and there is still a risk of reinfection, this paper uses the SIRS model and improves the SIRS model by combining the characteristics of financial risk contagion to study the risk contagion mechanism among big fintech companies.

### 2.2 Assumption of variables and parameters

#### 2.2.1 Three states of big fintech companies

The population is divided into three categories by the SIRS model: the susceptible, the infective, and the recovered. The recovered only have momentary immunity, and transition back to being susceptible after a certain period of time, making them susceptible to reinfection. When the susceptible come into touch with the ill, they become infectious and revert to the infective state. The infective can be treated and revert to the susceptible state with temporary immunity. The risk states given by big fintech companies during the risk contagion process are divided into three categories, and the existing SIRS model is improved by combining the characteristics of financial risk contagion.

1. Susceptible state: It refers to the state of being at risk of exposure and being susceptible to infection through contact with infected companies. Let S(t) represent the percentage of financial institution nodes that have not been infected at time t, 0≤ S(t)≤ 1.
2. Infective state: The state in which the risk has been infected and is likely to be transmitted to other financial institutions. Let I(t) represent the percentage of financial institutions nodes that are risk-infected and contagious at time t, 0≤ I(t)≤ 1.
3. Recovered state: The state in which a short-time immunity is obtained after the risk is removed. Let R(t) represent the percentage of financial institutions nodes that have been removed from the infected state at time t, 0≤ R(t)≤1.

#### 2.2.2 Assumption of parameters

The parameters of the model are assumed as follows:

1. Risk transmission rate (α). It is probability that a big fintech company that is vulnerable to infection is likely to transition to infection after the risk of infection. Parameter α is mainly affected by business relevance and public expectations.
2. Suppose that the cure rate is β. This refers to the probability that big fintech companies in the infected state will move out of the risk state and into the immune state through risk management or other means.
3. Assume that the direct immunization rate is γ. This represents the probability of directly acquiring immunity by taking timely and effective measures to prevent the risk before infection.
4. Considering that the big fintech companies infected with risks did not go bankrupt due to their financial strength, market position, and other factors, and temporarily removed the risks after taking measures, but did not gain immunity, we added the parameter δ to the conventional SIRS model to improve it, so that the model is more applicable to the risk contagion mechanism of big fintech.
5. Suppose that the immunity loss rate is ε. This refers to the probability that big fintech companies that have acquired immunity will lose their immunity due to new risks or the failure of original prevention measures, and transform into a vulnerable state.

**Fig 2:**
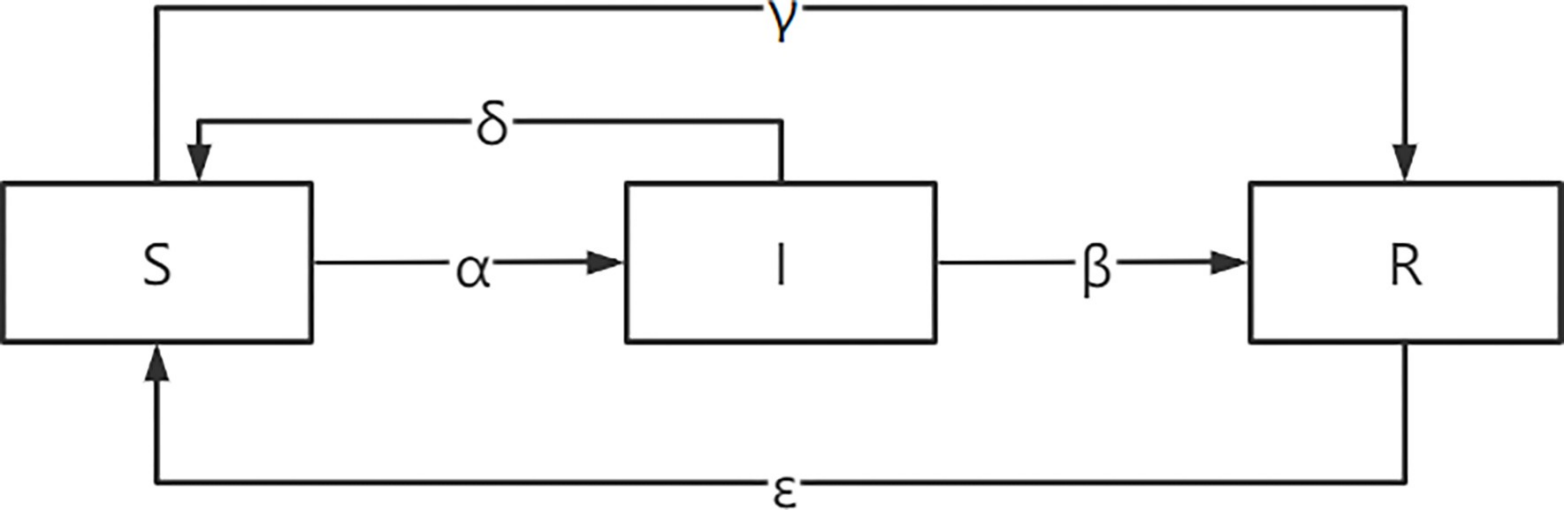
Risk contagion mechanism of big fintech.

According to the previous assumptions, *S*(*t*)+*I*(*t*)+*R*(*t*) = 1. Under the condition that the total number of nodes remains unchanged, the contagion mechanism of big fintech risk is shown in Fig 2.

### 2.3 Model construction

The following differential equation model is built using the aforementioned big fintech risk contagion mechanism and system dynamics modeling concept:

$$\left\{ \begin{array}{l} \frac{dS(t)}{dt}=-\alpha S(t)I(t)-\gamma S(t)+\delta I(t)+\varepsilon R(t)\\ \frac{dI(t)}{dt}=-\delta I(t)-\beta I(t)+\alpha S(t)I(t)\\ \frac{dR(t)}{dt}=-\varepsilon R(t)+\beta I(t)+\gamma S(t) \end{array}\right.$$ (1)

Substituting S(t)+I(t)+Rt) = 1 into Eq (1), we obtain:

$$\left\{ \begin{array}{l} \frac{dS(t)}{dt}=-\alpha S(t)I(t)-\gamma S(t)+\delta I(t)+\varepsilon [1-S(t)-I(t)]\\ \frac{dI(t)}{dt}=-\delta I(t)-\beta I(t)+\alpha S(t)I(t) \end{array}\right.$$ (2)

When the system equations reach a steady state, there are:

$$\left\{ \begin{array}{c} \frac{dS(t)}{dt}=0\\ \frac{dI(t)}{dt}=0 \end{array}\right.$$

That is:

$$\left\{ \begin{array}{l} -\alpha S(t)I(t)-\gamma S(t)+\delta I(t)+\varepsilon [1-S(t)-I(t)]=0\\ -\delta I(t)-\beta I(t)+\alpha S(t)I(t)=0 \end{array}\right.$$ (3)

Thus, the equilibrium point of Eq (3) can be obtained as follows:

$$E_{0}=\left(\frac{\varepsilon }{\gamma +\varepsilon },0\right),\;E_{1}=(\frac{\beta +\delta }{\alpha },\frac{\alpha \varepsilon -(\gamma +\varepsilon )(\beta +\delta )}{\alpha (\delta +\varepsilon )})$$

### 2.4 Basic regeneration number analysis

The basic reproduction number (R) represents the threshold of big fintech risk contagion and is the total number of big fintech companies that were infected during the entire period of financial risk contagion. It can also be simplified as the total number of big fintech companies that could be infected by a single big fintech company that was exposed to risk. The equilibrium point and the fundamental reproduction number of Eq (1) are as follows:

$$R=\frac{\alpha \varepsilon }{\left(\gamma +\varepsilon )(\beta +\delta \right)}$$

When R<1, the maximum number of infections is less than 1, and the risk tends to disappear. At this time, because I<0, it is out of its range, so Eq (3) has a unique equilibrium point E_0_.

When R>1, the maximum number of contagions exceeds 1, the risk contagion will always exist and eventually evolve into a crisis in the financial system. At this time, I_1_>0, E_0_ and E_1_ are the equilibrium points of Eq (3), E_0_ means that the risk tends to die out, E_1_ means that the risk has a diffusion trend.

### 2.5 Stability analysis of equilibrium points of the SIRS model

Next, we solve the equilibrium point of the system and analyze the stability of the system at the equilibrium point.

#### 2.5.1 Stability analysis of equilibrium point E_0_

The Jacobian matrix of Eq (2) at the equilibrium point $E_{0}=(\frac{\varepsilon }{\gamma +\varepsilon },0)$ is as follows:

$$J(E_{0})=\left[\begin{array}{cc} -(\gamma +\varepsilon ) & -\frac{\alpha \varepsilon }{\gamma +\varepsilon }+(\delta -\varepsilon )\\ 0 & -(\delta +\beta )+\frac{\alpha \varepsilon }{\gamma +\varepsilon } \end{array}\right]$$

The matrix has two eigenvalues: $\lambda_{1}=‐(\gamma +\varepsilon ),\;\lambda_{2}=‐(\delta +\beta )+\frac{\alpha \varepsilon }{\gamma +\varepsilon }$

The characteristic equation has a negative eigenvalue when R<1, λ_1_<0 and λ_2_<0, and it is locally stable at equilibrium point E_0_.

#### 2.5.2. Stability analysis of equilibrium point E_1_

The Jacobian matrix of Eq (2) at the equilibrium point $E_{1}=(\frac{\beta +\delta }{\alpha },\frac{\alpha \varepsilon ‐(\gamma +\varepsilon )(\beta +\delta )}{\alpha (\delta +\varepsilon )})$ is as follows:

$$J(E_{1})=\left[\begin{array}{cc} \frac{-\alpha \varepsilon +(\gamma +\varepsilon )(\beta +\varepsilon )}{\delta +\varepsilon } & -(\beta +\varepsilon )\\ \frac{\alpha \varepsilon -(\gamma +\varepsilon )(\beta +\delta )}{\delta +\varepsilon } & 0 \end{array}\right]$$

Consequently, the following is the characteristic equation of this matrix:

$$\lambda \left[\lambda -\frac{-\alpha \varepsilon +(\gamma +\varepsilon )(\beta +\varepsilon )}{\delta +\varepsilon }\right]+(\beta +\varepsilon )\frac{\alpha \varepsilon -(\gamma +\varepsilon )(\beta +\delta )}{\delta +\varepsilon }=0$$

From Veda’s theorem, we get:

$$\lambda_{1}+\lambda_{2}=\frac{-\alpha \varepsilon +(\gamma +\varepsilon )(\beta +\varepsilon )}{\delta +\varepsilon }$$

$$\lambda_{1}\lambda_{2}=(\beta +\varepsilon )\frac{\alpha \varepsilon -(\gamma +\varepsilon )(\beta +\delta )}{\delta +\varepsilon }$$

The characteristic equation’s eigenvalues are all negative for R>1, λ_1_+λ_2_<0, λ_1_λ_2_>0, and the equation is asymptotically stable at the equilibrium point E_1_.

According to the above analysis, when R<1, there is a risk extinction point E_0_ and it is asymptotically stable; When R>1, there is a risk contagion point E_1_ and it is asymptotically stable. Therefore, R = 1 is the threshold for risk contagion if and only if R>1, the risk contagion effect will be formed, and big fintech risks will spread in the system. A higher R means the risk will persist. Thus, controlling the size of R is the key to risk governance in big fintech. Because:

$$R=\frac{\alpha \varepsilon }{\left(\gamma +\varepsilon )(\beta +\delta \right)}$$

Therefore, when other factors remain unchanged, the smaller α and ε are, the smaller *R* is. The larger γ, β and δ are, the larger R is. The fourth section will simulate the spread of the risk when the influencing factors of the big fintech risk are intervened by adjusting the size of the above parameters.

## 3 Result and discussion

In this section, the stated ordinary differential equations are simulated using the MATLAB 2019 data modeling tool. The simulation focuses on the change of parameters and creates a big fintech risk infection scenario.

### 3.1 The density change of different nodes at the initial time

Based on the parameter assumptions of the SIRS model described in the preceding section, on the premise that the basic regeneration number R>1, this paper sets the proportion of big fintech companies and the parameter values of each state node at the initial time as follows: The density of big fintech companies at risk exposure S(0) = 0.9, the density of big fintech companies at risk of infection I(0) = 0.05, the density of big fintech companies at immune to risk R(0) = 0.05, α = 0.6, β = 0.15, γ = 0.1, δ = 0.15, ε = 0.3, the observation time is two months (t ≤ 60). At the same time, the density of each node in the initial state is adjusted to facilitate comparison: S(0) = 0.7, I(0) = 0.15, R(0) = 0.15, and the other parameters remain unchanged to observe the change of risk contagion.

**Fig 3:**
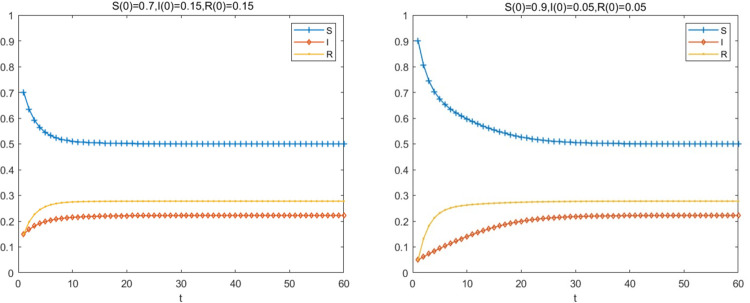
Density of nodes in initial state.

The simulation results are shown in Fig 3. As can be seen from Fig 3, the density of big fintech companies in the infected state shows an increasing trend over time. The higher the density of companies in the infected state at the initial moment, the faster the spread, and the shorter the time it takes for the number of infections to peak. This is because the greater the number of companies in the infected state, the more susceptible state companies are contacted, and the greater the effective contact rate. After reaching the peak, the density of infected big fintech companies does not show a decreasing trend, indicating that the risk is still spreading in the connected network at this time.

### 3.2 Impact of infection rate change on risk transmission process

**Fig 4:**
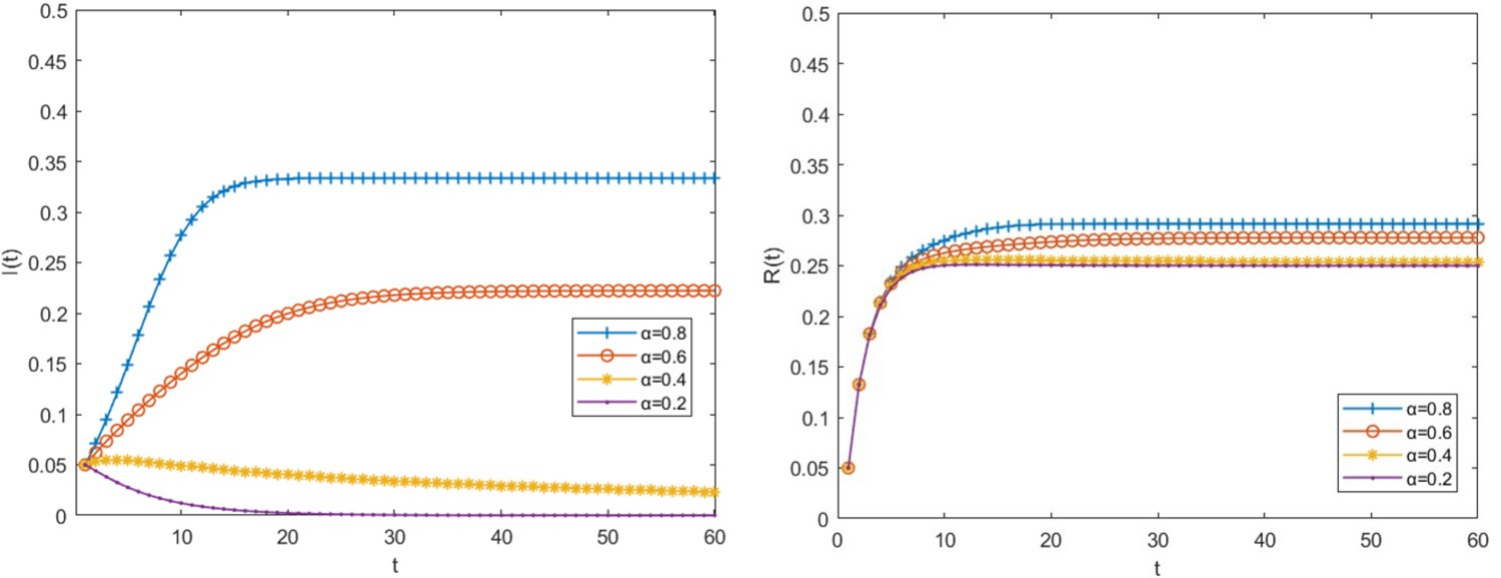
The impact of the risk contagion rate α on the risk transmission process.

Other parameter values remain unchanged when the risk transmission rate α is 0.8,0.6,0.4 and 0.2 respectively, the density changes of big fintech companies in the infected state and immune state are shown in Fig 4.

It can be seen from Fig 4 that as the risk transmission rate decreases, the density of risk-infected big fintech companies in the relational network is also decreasing. The higher the transmission rate, the faster the density of risk-infected companies peaks, meaning that the big fintech risk has a greater impact on other financial institutions. When α is reduced to 0.4, the density of infected big fintech companies will reach the peak on the fourth day, and then it will decline rapidly, but it does not tend to zero, which means that the risk has not been resolved. When drops to 0.2, the basic regeneration number R<1, the risk contagion threshold is not exceeded, the density of infected companies gradually approaches zero as time goes on, and the risk is essentially resolved after the 20th day. As a result, when the system runs as intended, the spread of risk will gradually be repressed as the transmission rate decreases, and the amount of time needed to resolve the risk will get shorter and shorter.

### 3.3 Impact of cure rate change on risk transmission process

**Fig 5:**
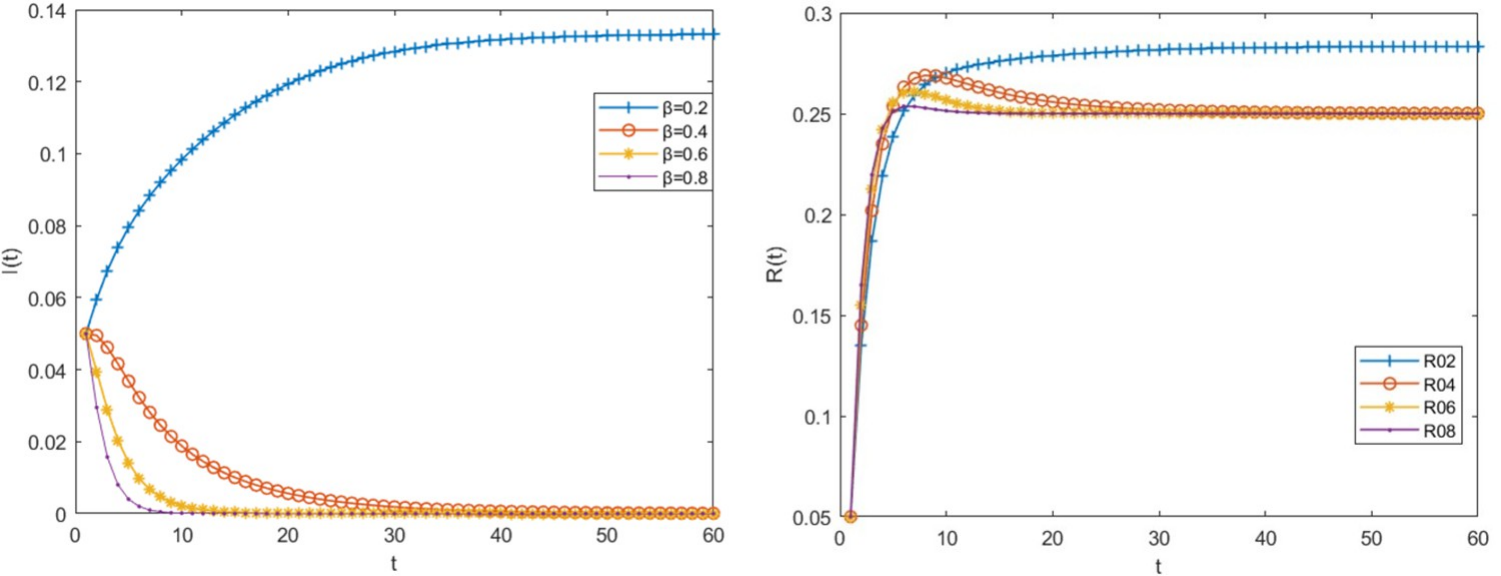
The impact of the cure rate β on the risk transmission process.

Other parameter values remain unchanged when the cure rate β is taken as 0.2,0.4,0.6 and 0.8 respectively, the density changes of big fintech companies in infectious and immune status are shown in Fig 5.

As can be seen from Fig 5, when β = 0.2, the basic regeneration number R>1, it indicates that big fintech companies with infection risk lack risk governance ability or have weak risk governance ability, and cannot take effective measures to resolve the risk after being infected. Therefore, the number of big fintech companies with risk is increasing. When β is 0.4, 0.6, 0.8, at this point R<1, it indicates that the company has launched certain emergency management measures at this time, successfully avoiding dangers and making the emergency a safe haven. The larger β is, the higher the level of risk governance of the company is, the stronger its anti-risk ability is, the faster the number of infected big fintech companies decreases, and the shorter the time to resolve risks is. However, there was little change in the number of permanently immune companies(R(t)). This is owing to the fact that the big fintech companies that survived the crisis were only ex-post remedial after being exposed to the risk, making them only momentarily immune and susceptible to infection after coming into touch with other exposed financial institutions.

### 3.4 Impact of direct immunization rate change on risk transmission process

**Fig 6:**
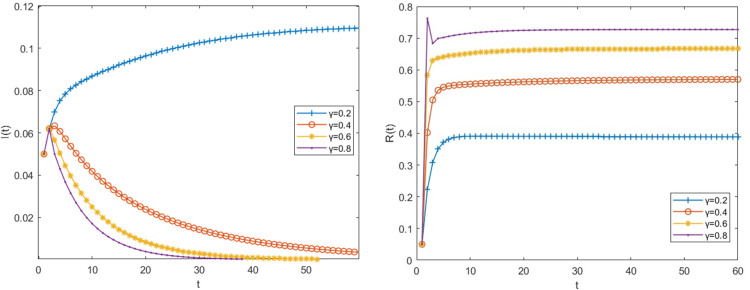
The impact of the direct immunization rate on the risk transmission process.

Other parameter values remain unchanged when the direct immunity rate γ is taken as 0.2, 0.4, 0.6 and 0.8 respectively, the density changes of big fintech companies in the infected state and immune state are shown in Fig 6. The direct immunity rate reflects the risk prevention level of the company, that is, the company is not infected by the risk based on effective defense measures before the risk infection, and directly obtains the immunity to the risk. Fig 6 illustrates this as follows:

(1) When γ = 0.2, the basic regeneration number R>1, the number of companies infected with risk rose rapidly over the observed period, indicating the spread of risk in the network. When γ is equal to 0.4,0.6,0.8, R<1, the number of companies in the infected state is decreasing over time. When γ = 0.6 and 0.8, the number of big fintech companies in the infected state(I(t)) gradually tends to 0, indicating that the risk will ultimately be eliminated. The number of big fintech companies in the immune condition will reach its peak and then stabilize, the higher the value of γ, the faster the growth rate. This demonstrates that companies with high direct immunity rates can be protected against infection by taking effective risk prevention measures "in advance" of a risky event. As a result, the potential for risk contagion can be somewhat reduced.

(2) The enhancement of the direct immunization rate has a bigger impact on the company’s ability to gain risk immunity than the cure rate does. As a result, preventative action is preferable to responding to emergencies.

### 3.5 Impact of probability of not acquiring immunity change on risk transmission process

**Fig 7:**
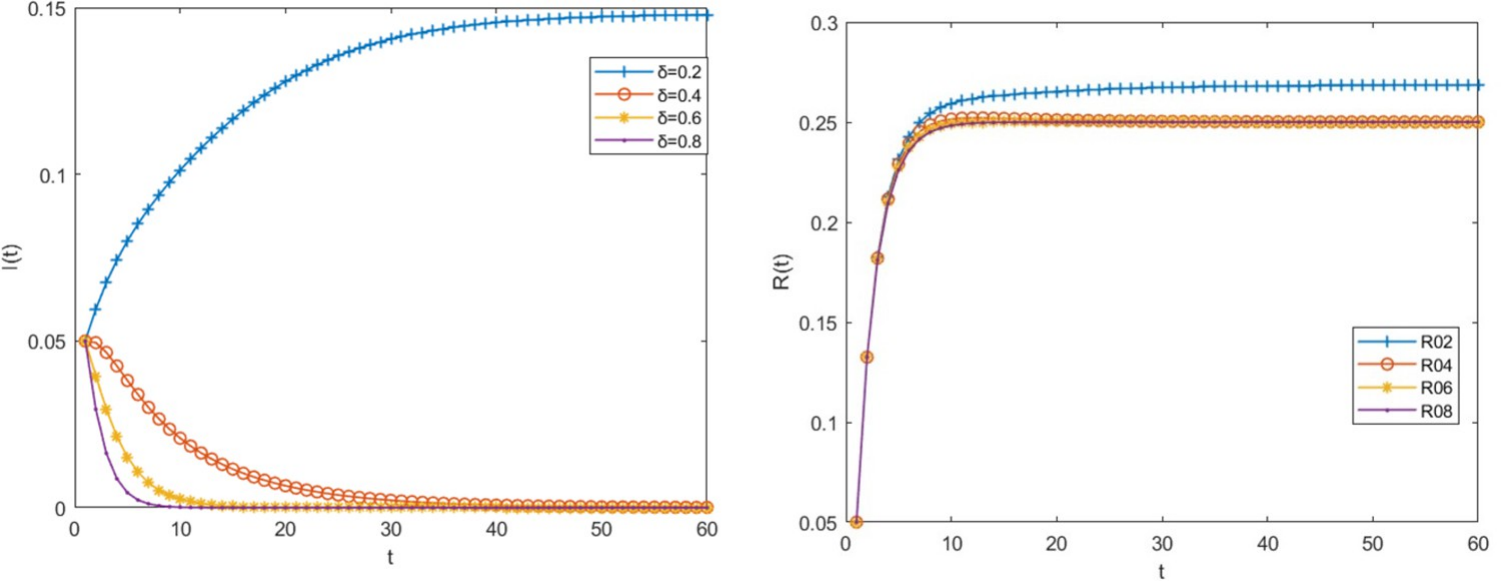
The impact of probability of not acquiring immunity on the risk transmission process.

The other parameter values remain unchanged when the probability δ that the big fintech companies in the infected state do not acquire the immune ability and become susceptible to infection is 0.2, 0.4,0.6 and 0.8 respectively, the density changes of big fintech companies in the infected state and immune state are shown in Fig 7. It can be seen from Fig 7 that although δ can reduce the density of infected big fintech companies, it is a way based on the fact that the big fintech company has not really resolved the risk, and its potential impact will be detrimental to the big fintech company’s operation and sustainable development. Therefore, this way is not recommended in reality to move the company out of the infected state.

### 3.6 Impact of the immune loss rate change on risk transmission process

**Fig 8:**
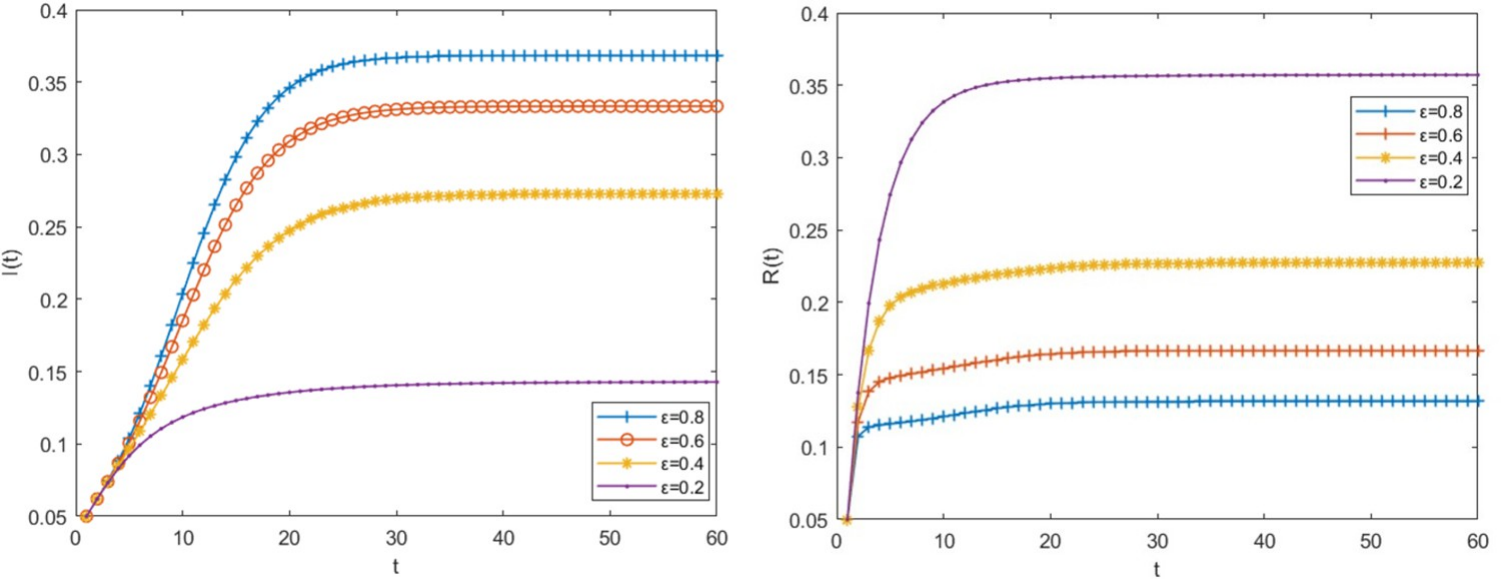
The impact of the immune loss rate on the risk transmission process.

When other parameter values remain unchanged, the immunity loss rate ε is 0.8, 0.6, 0.4 and 0.2 respectively, the density changes of big fintech companies in the infected state and immune state are shown in Fig 8. It can be seen from Fig 8 that the lower the loss rate of immunity is, the higher the probability that the big fintech companies have acquired immunity will stay in the immune state rather than return to the susceptible state, and the proportion of the number of big fintech companies in the infected state will also continue to decrease, thus inhibiting the spread of risks in the relational network.

## 4 Conclusions

This article demonstrates the law of risk contagion among big fintech companies by constructing the SIRS epidemic model. From the aforementioned simulation research, it can be concluded that:

First, reducing the risk transmission rate α and immunity loss rate ε, and increasing the cure rate β, direct immunity rate γ and the probability of not acquiring immunity δ, that is, enhancing the risk warning, risk management, risk buffering and blocking capabilities of big fintech companies, and timely improving risk prevention measures in the post-infection phase, with time going by, I(t) will gradually tend to 0, R(t) will gradually tend to be stable, and the model will gradually approach the risk extinction equilibrium point E_0_ and achieve progressive stability, it means that the big fintech risk is controlled.

Second, infectious effects do not necessarily occur when risks arise. Only when the basic regeneration number R exceeds the threshold for risk contagion, that is, when the number of big fintech companies infected with the risk exceeds the risk threshold, the big fintech risk will spread in the New Relational network.

Third, proactive interventions are more effective than post-incident response measures.

As a result, preventing and eliminating big fintech risks should begin in three stages: before, during, and following the event. The following recommendations are made in this paper in light of the findings of previous research.

### 4.1 Establishing risk monitoring and early warning mechanism to improve the direct immunization rate of Big fintech companies

Fintech has accelerated the spread and pace of financial risks while fostering the quick development of financial innovation. It has also made supervision more challenging. In order to effectively prevent and control financial risks in advance and increase the direct immunity, big fintech companies should actively use regulatory technology to build a risk monitoring model that is consistent with the situation of financial institutions. They should also timely handle the corresponding risks through intelligent monitoring, comprehensive prediction, and real-time information submission at the early stage of risk spread. Additionally, establishing mechanisms for isolating and blocking financial risks, reducing risk transmission rates, and preventing cross-contamination of risks are essential.

### 4.2 Strengthening the ability of risk governance and enhance the ability of big fintech companies to cure risks

Big fintech companies play a crucial role in the risk transmission process; as such, if they are unable to effectively address the infected risk, it will not only be detrimental to their long-term development but will also cause the risks to spread or even spill over. Therefore, big fintech companies should establish scientific and standardized risk management systems, strengthen the introduction and training of regulatory talent, apply new technologies, improve the risk resolution measures, and prevent the scenario where big fintech companies are not fully resolved risks and are exposed to risks again.

### 4.3 Improving the internal and external supervision mechanism to promote the sustainable development of enterprises

Risk resolution is temporary for big fintech companies which have obtained immunity, and these companies will still be vulnerable to risks due to the failure of existing measures or the emergence of new risks. Therefore, from the perspective of big fintech companies, in addition to strengthening their risk governance ability, they should also grasp the policy trend in time and conduct self-inspection and correction. These actions will help them achieve sustained immunity and stop the further spread of risks.

## References

[1] Alt R., Beck R., Smits M.T. FinTech and the transformation of the financial industry. Electron Markets. 2018; 28: 235–243.

[2] Yin Cheng. Logics and Suggestions of BigTech in Financial Infrastructure Construction. The Chinese Banker (in Chinese), 2020; 2: 70–72. doi: 10.3969/j.issn.1671-1238.2020.02.022

[3] World Economic Forum (Forum), Deloitte. Beyond Fintech: A pragmatic assessment of disruptive potential in financial services, 2017; Available from: https://www3.weforum.org/docs/Beyond_Fintech_-_A_Pragmatic_Assessment_of_Disruptive_Potential_in_Financial_Services.pdf

[4] Frost J, Gambacorta L, Huang Y, Shin HS, Zbinden P. BigTech and the changing structure of financial intermediation. Economic Policy, 2019; 34: 761–799.

[5] Basel Committee on Banking Supervision (BCBS). Sound Practices: Implications of Fintech Developments for Banks and Bank Supervisors, 2017; Available from: https://www.bis.org/bcbs/publ/d431.htm

[6] Wen Shigang, Li Jianping, Huang Chuangxia, Zhu Xiaoqian. Extreme risk spillovers among traditional financial and FinTech institutions: A complex network perspective. The Quarterly Review of Economics and Finance. 2023; 88: 190–202.

[7] Chaudhry Sajid M., Ahmed Rizwan, Toan Luu Duc Huynh, Chonlakan Benjasak. Tail risk and systemic risk of finance and technology (FinTech) firms. Technological Forecasting and Social Change. 2022; 174:121191.

[8] Ahern D. Regulatory Lag. Regulatory Friction and Regulatory Transition as FinTech Disenablers: Calibrating an EU Response to the Regulatory Sandbox Phenomenon. Eur Bus Org Law Rev. 2021; 22: 395–432.

[9] Lee In, Yong Jae Shin. Fintech: Ecosystem, business models, investment decisions, and challenges. Business Horizons. 2018; 61: 35–46.

[10] Gai Keke, Qiu Meikang, Sun Xiaotong. A survey on FinTech. Journal of Network and Computer Applications. 2018; 103: 262–273.

[11] Liu. Fintech’s Main Functions, Risk Characteristics and Normative Regulation. South China Finance (in Chinese). 2021; 10: 63–71. doi: 10.3969/j.issn.1007-9041.2021.10.006

[12] Financial Stability Board. Financial Stability Implications from FinTech. 2017. Available from: https://www.fsb.org/2017/06/financial-stability-implications-from-fintech/

[13] Li. Integration of Finance and Technology: Definition, Determinants and Risks. International Economic Review (in Chinese). 2020; 3: 91–106+6.

[14] Boissay, Frédéric, Ehlers T., Gambacorta L., & Shin H. S. Big techs in finance: on the new nexus between data privacy and competition. Social Science Electronic Publishing,2021.

[15] Waal MD, Poell T., Dijck J. V. The Platform Society. Public Values in a Connective World, Oxford: Oxford University Press; 2018.

[16] Financial Stability Board. BigTech firms in finance in emerging market and developing economies. 2020. available from: https://www.fsb.org/2020/10/bigtech-firms-in-finance-in-emerging-market-and-developing-economies/

[17] Yin. Feng, BigTech in Finance: Definition, Development and Challenges. Chinese Review of Financial Studies (in Chinese). 2020; 03: 65–75+125.

[18] Financial Stability Board. BigTech in finance: Market developments and potential financial stability implications. 2019. available from: https://www.fsb.org/2019/12/bigtech-in-finance-market-developments-and-potential-financial-stability-implications/

[19] Yi Fang, Qi Wang, Mounyan Zhang. Hidden financial risks and Regulation of big Tech companies. Study and Practice (in Chinese). 2021; 8: 54–66. doi: 10.19624/j.cnki.cn42-1005/c.2021.08.006

[20] Valverde S. C. and Fernandez F. R. Financial Digitalization: Banks, Fintech, Bigtech, and Consumers. Journal of Financial Management, Markets and Institutions. 2020; 8(1): 1–13.

[21] May R M, Levin S A, Sugihara G. Ecology for bankers. Nature. doi: 10.1038/451893a 18288170

[22] Garas A., Argyrakis P., Rozenblat C., Tomassini M., Havlin S. Worldwide spreading of economic crisis. New Journal of Physics. 2010; 12(2): 185–188.

[23] Toivanen M. Contagion in the interbank network: an epidemiological approach. Ssrn Electronic Journal. 2013. doi: 10.2139/ssrn.2331300

[24] Research Group of PBC Guangzhou Branch. Research on Financial Risk Prevention and Control Based on Complex Network Infectious Disease Model. South China Finance (in Chinese). 2021; 7: 29–39. doi: 10.3969/j.issn.1007-9041.2021.07.002

[25] Haldane A. Rethinking the financial network. Fragile Stabilität–stabile Fragilität. 2013; 53: 243–278.

[26] Research Group of PBC Nanning Branch. Research on risk cross-contagion mechanism and prevention and Control strategy of financial market—Based on SIRS Infectious Disease Model. South China Finance (in Chinese). 2017; 2: 3–13. doi: 10.3969/j.issn.1007-9041.2017.02.001

[27] Wang, Tan, Chen, The Contagion Mechanism of Default Public Opinion in P2P Network Lending Creditor’s Rights Market. Finance Forum (in Chinese), 2017, (11),56–69. doi: 10.16529/j.cnki.11-4613/f.2017.11.007

[28] Xu Yu. Research on the Application of Risk Contagion Model of Mutual Guarantee Financing for SMEs Cluster. Accounting Research. 2018; 1: 82–88. doi: 10.3969/j.issn.1003-2886.2018.01.013

[29] Mi Qian. Internet financial risk contagion based on SEls model. Journal of Nanjing University of Science and Technology, 2019; 43(06):800–806. doi: 10.14177/j.cnki.32-1397n.2019.43.06.019

[30] Magnuson W. "Regulating Fintech.". Vanderbilt Law Review. 2018; 71(4):1167–1226.

[31] Chakravorti S. "Analysis of Systemic Risk in Multilateral Net Settlement Systems." Journal of International Financial Markets, Institutions and Money. 2000; 10(1):9–30.

[32] Brynjolfsson E., MD Smith. Frictionless commerce? a comparison of internet and conventional retailers. Management Science. 2000; 46 (4):563–585.

[33] Castrén, & Kavonius I. K. Balance sheet interlinkages and macro-financial risk analysis in the euro area. ECB Working Paper No.1124. 2009. Available from: https://www.ecb.europa.eu/pub/pdf/scpwps/ecbwp1124.pdf

[34] Rodriguez Juan Carlos. Measuring financial contagion: A Copula approach. Journal of Empirical Finance. 2007; 14(3):401–423.

[35] GILBERTE, KARAHALIOS K. Widespread worry and the stock market ll Proceedings of the Fourth International AAAl Conference on Weblogs and Social Media. Washington, DC: AAAl Press. 2010; 58–65.

[36] Billio M., Lo A. W., Sherman M. G., Pelizzon L. Econometric measures of connectedness and systemic risk in the finance and insurance sectors. Social Science Electronic Publishing. 2012; 104(3): 535–559.

